# Editorial

**Published:** 1958-07

**Authors:** 


### *Editorial*

The price of our recently acquired ability to treat infections by antibiotics is the emergence of the drug resistant organism. The "hospital staphylococcus" is of course a direct result of such therapy and there is no doubt that it is found most commonly in those hospitals and wards in which antibiotics are most freely used. As Americans say, the problem of staphylococcal sepsis is now common "in all medically advanced parts of the world"[1]. Disquieting and worrying as are these epidemics due to the hospital staphylococcus and other resistant organisms they are also proving to be of some value; for as a result, we are being forced to study anew the essential epidemiology of such infections. We are being made to think again and to think hard about the way in which these organisms spread from case to case and from carrier to carrier and how they gain access to tissues. We have learnt that, as is so often the case, for every infection produced there are large numbers of symptomless carriers. The work of Dr. Gillespie and others has shown the frequency with which nasal carriers occur and how quickly the nares can become infected. Yet at the same time it would appear that although these nasal carriers may spread the organism they are not commonly the source of serious infections. The reason for this anomalous state of affairs is still to be found; in fact our knowledge of this aspect of the problem, which might be called the ecology of the staphylococcus, is all too meagre. In this number Dr. Gillespie[2] describes some of the slow and laborious steps by which these gaps in our knowledge are being filled. Outbreaks of serious infections with these organisms seem usually to be related to the presence of staphylococcal sepsis—infected wounds or boils—in the community. All the studies which have been made on the problem, as for example those of Gillespie and of Barber and Dutton[3], serve to remind us forcibly that staphylococcal sepsis is an infectious disease which should be regarded with respect and isolated as carefully and as efficiently as possible. While it is clear that these resistant organisms only assume prominence as a cause of infection in communities in which antibiotics are widely and perhaps excessively used it is also clear that it is now too late to prevent their ravages by reducing our use of antibiotics. Nevertheless it is important that we should remember that organisms which show resistance to one of the groups of antibiotics very rapidly become resistant to the others and it is urgently necessary for us to reserve the newer antibiotics for serious infections with these resistant organisms.

Another value of the outbreaks of infection with resistant organisms in our hospitals is that they are serving to remind us that our sometimes vaunted "asepsis" is only as strong as its weakest point and that this is often very weak. We can no longer rely on efficient asepsis alone, but must be ready to supplement this at any time with intelligent use of antiseptics while we are searching for the break in the aseptic chain which has permitted infection to spread.

C.B.P.

1 *J. Amer. med. Ass.*, 1958. 166. 1177–1203.

2 Page 56.

3 *Lancet*, 1958. II 64.

